# Experimental and Numerical Evaluations of Localized Stress Relaxation for Vulcanized Rubber

**DOI:** 10.3390/polym14050873

**Published:** 2022-02-23

**Authors:** Kijvanish Sukcharoen, Nitikorn Noraphaiphipaksa, Anat Hasap, Chaosuan Kanchanomai

**Affiliations:** 1 Department of Mechanical Engineering, Faculty of Engineering, Thammasat University, Pathumthani 12120, Thailand; sukcharoen@engr.tu.ac.th (K.S.); noraphaiphipaksa@gmail.com (N.N.); 2 Nitikorn Research Partner Co., Ltd., Lumlukka, Pathumthani 12150, Thailand; 3 Railway Transportation System Testing Center, Thailand Institute of Scientific and Technological Research, Pathumthani 12120, Thailand; anat@tistr.or.th

**Keywords:** stress relaxation, localized stress, viscoelastic, rubber, finite element analysis

## Abstract

Vulcanized rubbers are commonly used to provide the energy absorption under compressive deformation from other engineering components. However, if a constant compressive deformation is maintained on rubber, the load response is not constant but decreases with time; i.e., the stress relaxation. A decrease in force response with time of rubber can be experimentally evaluated by the stress relaxation test. In the present work, the localized stress of vulcanized rubber during a compressive stress relaxation test (i.e., ASTM D6147) was evaluated. Hyperelastic behavior was assumed during rapid application of strain, while the viscoelastic behavior was assumed during stress relaxation. Hyperelastic and viscoelastic parameters were experimentally evaluated using a standard specimen. Finite element analysis (FEA) models were applied for the predictions of stress relaxations of rubbers with various geometries and applied strains. FEA results were in good agreement with results of the stress relaxation tests. Localized stresses in rubber during rapid application of compressive strain and stress relaxation were successfully evaluated. The findings can give the localized phenomena of vulcanized rubber during a stress relaxation test, which can be used as a guideline for the design, usage, and improvement of rubber and viscoelastic polymeric components.

## 1. Introduction

Vulcanized rubbers are commonly used to provide the energy absorption under compressive deformation from other engineering components. They can be operated under large deformation from compressive load, and recovers to the original dimension when the compressive load is removed. However, if a constant compressive deformation is maintained on rubber, the load response is not constant but decreases with time; i.e., the stress relaxation [1]. During assembly of engineering components, the compressive deformation of rubber can be induced from the weight of the components as well as the assembled load. With the increase in operation period, the tightness of the assembly may be compromised by the stress relaxation of rubber.

A decrease in force response with time of rubber can be experimentally evaluated by the stress relaxation test. Under the jurisdiction of ASTM Committee on Rubber, as well as ASTM Subcommittee on Time and Temperature-Dependent Physical Properties, the ASTM D6147 [2] has been proposed for determining the force decay of vulcanized rubber or thermoplastic elastomer under a constant compressive deformation. Based on ASTM D6147 recommendations, the compression of a specimen to the specified value should be completed within 30 s. Subsequently, the deformation is maintained constant, while the reduction in force response with time was measured.

Under rapid application of compressive strain, the stress–strain relationship of rubber is assumed to be non-linearly elastic, isotropic, and incompressible. As a constitutive model derives from a strain energy density function, the hyperelasticity can be applied to characterize the stress–strain behavior of rubbers. Many hyperelastic models have been proposed using phenomenological descriptions; e.g., the Mooney–Rivlin model [3], Ogden model [4], and Yeoh model [5]. These hyperelastic models were successfully applied in various applications. Karen et al. [6] performed experiments to define hyperelastic and viscoelastic models for non-linear finite element simulations of rubber components. The stiffness curves of the different shapes of designs were numerically estimated, and the best curve was chosen as an optimum design solution. Bradley et al. [7] showed that the Ogden strain-energy density function can be used to provide planar stress–strain data when only uniaxial data are available. Kim et al. [8] performed the uni-axial tension test, biaxial tension test, and pure shear test on automotive transmission rubber mounts, and characterized the results using the Mooney–Rivlin model. The dynamic stiffness of an automotive transmission rubber mount was predicted with finite element analysis. Predictions were close to the experimental results. Ghoreishy [9] selected four hyperelastic constitutive equations, combined with the Prony equation to describe the hyper-viscoelastic behavior of a rubber compound under uniaxial tension. These Prony parameters were used for the simulation of other samples to show the applicability and repeatability of the developed method.

On the other hand, when deformation is maintained constant, the rubber is likely to exhibit time-dependent behavior or viscoelasticity (i.e., the stress relaxation). Various viscoelastic models have been proposed (e.g., the Maxwell model [10] and Kelvin–Voigt model [11]) and used in many research works [12,13,14,15,16]. Sorvari and Malinen [15] proposed a numerical method for computing the relaxation modulus of a linearly viscoelastic material. The method can be applied for times shorter than the half of time when the maximum strain is achieved. Martynova and Stetsenko [16] compared the relaxation functions using various methods: (i) the backward recurrence method; (ii) a method using formulas of numerical integration; and (iii) a method based on the prior assumption that the material behavior is described by the generalized Maxwell model. They found that these methods give similar results. Therefore, they suggested that the third method is preferable, because it is possible to immediately find the relaxation function in the form of a truncated Prony series from the finite element analysis (FEA) software.

Although there are many experimental and numerical works regarding the stress relaxation of viscoelastic materials (e.g., References [12,13,14,15,16]), most of them are emphasized on the bulk behavior of a specimen. However, the complex geometry of the actual component causes the localized stress responses, which are necessary for the design, usage, and improvement of rubber components (e.g., rail pad, O-ring, rubber bearing, car tires). Therefore, the localized stress of vulcanized rubber during a compressive stress relaxation test (ASTM D6147 [2]) was firstly evaluated in the present work.

The compressive stress relaxation tests were performed at various strains (10% to 50% strains). Rubber was assumed to be hyperelastic, isotropic, and incompressible under rapid application of compressive strain, while it was assumed to be viscoelastic during stress relaxation. To extend the benefit of the stress relaxation test of the standard specimen recommended by ASTM D6147 [2], the finite element analysis (FEA) models were used for the predictions of stress relaxations of rubbers with various geometries and applied strains. The FEA results were compared with the results of the stress relaxation tests for validation. Subsequently, the stress distributions in rubber as well as at contact with the compression plate during rapid application of compressive strain and stress relaxation were numerically evaluated using a validated FE model. The findings can give the localized phenomena of vulcanized rubber during rapid application of compressive strain and stress relaxation, which can be used for the design, usage, and improvement of rubber components.

## 2. Theory

**Figure 1:**
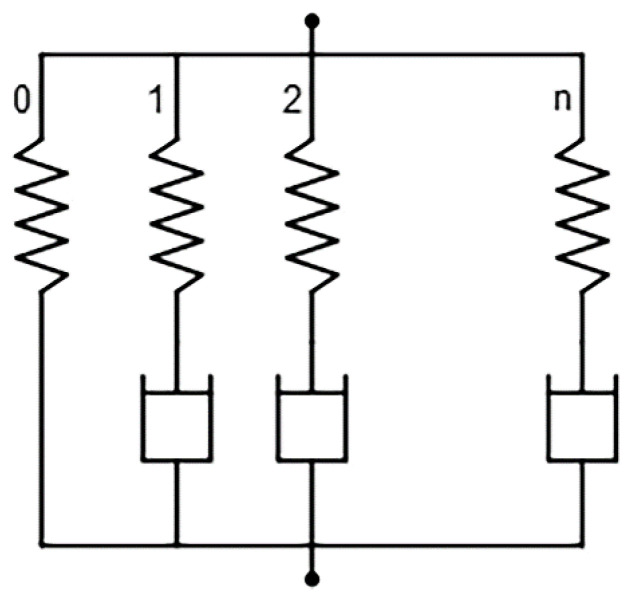
Model with multiple parallel hyperelastic and viscoelastic networks.

Based on the rapid application of compressive strain (i.e., ASTM D6147 [2]), the rubber was assumed to be non-linearly elastic, isotropic, and incompressible during rapid application of compressive strain; i.e., hyperelastic behavior. Subsequently, during constant applied deformation, the rubber was assumed to behave viscoelastically. The hyperelastic and viscoelastic behaviors of rubber were modeled by the parallel rheological framework (PRF). PRF consists of multiple parallel hyperelastic and viscoelastic networks (Figure 1). Similar definition of hyperelastic material was applied for all networks (0 to *n* network), while a different definition of viscoelastic material was applied for each network (1 to *n* network). A hyperelastic response is defined using the strain energy potential, while a viscoelastic response is defined using the flow rule derived from a creep potential. From the results of multiple stress relaxation tests, the hyperelastic and viscoelastic parameters were estimated using commercial FEA software (i.e., Abaqus/Standard [17]). Details of the modeling of the hyperelastic and viscoelastic behaviors are as follows.

### 2.1. Hyperelastic Behavior

For rubberlike material, the hyperelastic model provides a general strain energy potential (i.e., the strain energy stored in material per unit of reference volume as a function of strain) to describe the material behavior for nearly incompressible elastomer. This nonlinear elasticity model is valid for large elastic strain. Therefore, the present rubber was assumed to be isotropic and nearly incompressible, and then the strain energy potential could be formulated as a function of strain invariants. For nearly incompressible rubber, the Poisson’s ratio was assumed to be 0.495. As a special case of the reduced polynomial model, the Yeoh model [5] was used for the determination of strain energy potential (*U*), as follows:

(1) $$U=C_{10}\left(\bar{I_{1}}-3\right)+C_{20}{\left(\bar{I_{1}}-3\right)}^{2}+C_{30}{\left(\bar{I_{1}}-3\right)}^{3}+\frac{1}{D_{1}}{\left(J^{el}-1\right)}^{2}+\frac{1}{D_{2}}{\left(J^{el}-1\right)}^{4}+\frac{1}{D_{3}}{\left(J^{el}-1\right)}^{6}\;$$

where $C_{i0}$ and $D_{i}$ are the material parameters, $J^{el}$ is the elastic volume ratio, and ${\bar{I}}_{1}$ is the first deviatoric invariant, which is,

(2) $${\bar{I}}_{1}={\bar{\lambda }}_{1}{}^{2}+{\bar{\lambda }}_{2}{}^{2}+{\bar{\lambda }}_{3}{}^{2}\;$$

(3) $${\bar{\lambda }}_{i}=J^{-1/3}\lambda_{i}\;$$

where $\lambda_{i}$ are the principal stretches, and J is the total volume ratio. Without the influence from thermal expansion, the elastic volume ratio was assumed to be the total volume ratio. For the uniaxial deformation, the principal stretches ($\lambda_{i}$) are

(4) $$\lambda_{1}=\lambda_{U}=1+\varepsilon_{U}$$

(5) $$\lambda_{2}=\lambda_{3}=\frac{1}{\sqrt{\lambda_{U}}}\;$$

where $\lambda_{U}$ is the stretch in compressive direction, and $\varepsilon_{U}$ is the normal strain in compressive direction.

### 2.2. Viscoelastic Behavior

A viscous response from viscoelastic networks (network 1 to *n*) was defined by a Prony series expansion [11], as follows:

(6) $$\sigma \left(t\right)=Y\left(t\right)\cdot \varepsilon_{o}\;$$

where *σ*(*t*) is the stress in a function of time, *ε_o_* is the peak strain, and *Y*(*t*) is the relaxation function, which is defined as follows:

(7) $$Y\left(t\right)=E_{o}\left(1-\overset{n}{\underset{i=1}{\sum }}p_{i}\left(1-e^{-t/\tau_{i}}\right)\right)\;$$

The relaxation functions for initial stage (*t* = 0) and the complete relaxation stage (*t* = ∞) are as follows:

(8) $$Y\left(t=0\right)=E_{o}\;$$

(9) $$Y\left(t=\infty \right)=E_{o}\left(1-\overset{n}{\underset{i=1}{\sum }}p_{i}\right)\;$$

where, *p_i_* is the Prony constant, *τ_i_* is the Prony retardation time constant, and *E_o_* is the instantaneous modulus.

## 3. Material

**Figure 2:**
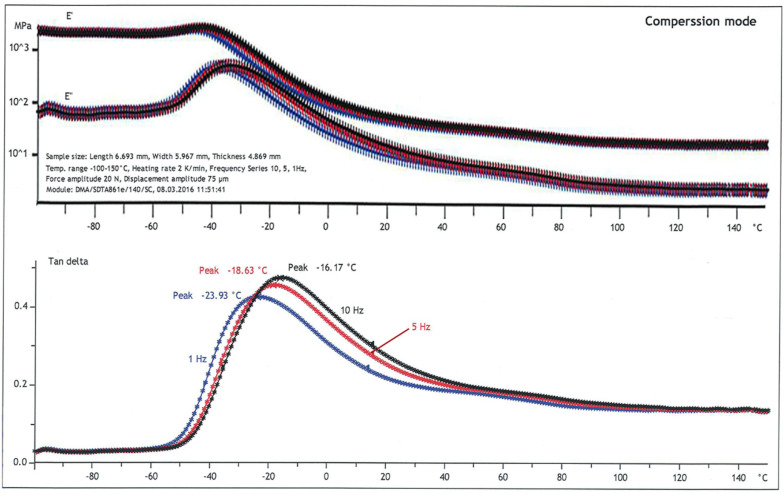
Dynamic mechanical analysis of rubber.

Nitrile butadiene rubber (NBR) was supplied in a sheet of 8-mm thickness. Based on ASTM D4065 [18], the dynamic mechanical analysis (DMA) was conducted in compression mode on a Mettler Toledo DMA/SDTA 861^e^. Multi-frequency experiments were performed by increasing the temperature (−100 to 150 °C) in ramp mode with 2 °C/min at 1, 5, and 10 Hz. The elastic compressive modulus (*E*′), viscous compressive modulus (*E*″), and damping coefficient (tan *θ* = *E*″/*E*′) are shown in Figure 2. The transition from the glassy state to the rubbery state (i.e., the drop in modulus with temperature) occurs at approximately −40 °C (i.e., glass-transition temperature or *T*_g_). The damping coefficient in the rubbery state depends on the testing frequencies; i.e., it increases with frequency.

## 4. Experiment

The stress relaxation test was performed in accordance with ASTM D6147 [2]. The rubber specimen was compressed to a constant deformation under a specified time, then the deformation was maintained constant. Variation in the counterforce with time was measured by a load cell and used for the calculation of the compressive stress response; i.e., the fraction between the counterforce and initial load bearing area. Stress response varied with compressive strain until the peak strain was reached; i.e., the hyperelastic period. Subsequently, the peak strain was maintained, and then the stress relaxed over time due to the viscous effects in rubber i.e., viscoelastic period.

**Figure 3:**
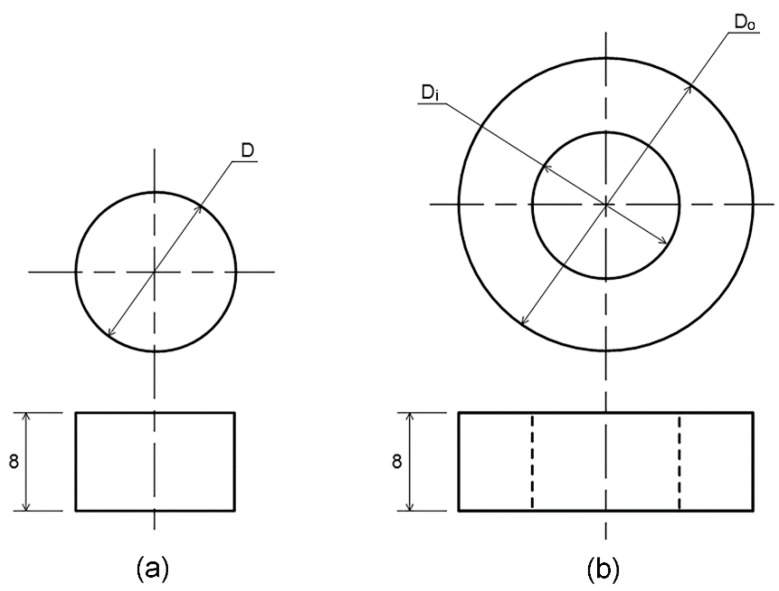
Geometries of the rubber specimen: (**a**) disc, and (**b**) doughnut (dimension in mm).

Three types of specimens (i.e., two discs and one doughnut) were prepared from a rubber sheet using cutting dies. Geometries of the specimen are shown in Figure 3, while the dimensions of specimen are listed in Table 1. As a non-dimensional ratio of contact area to stress free area, the shape factors at initial stage are also listed in Table 1. Constants of the hyperelastic and viscoelastic models were obtained from stress relaxation tests of disc-A; i.e., a standard specimen recommended by ASTM D6147 [2]. Subsequently, they were used for the numerical predictions of stress relaxations of disc-B and doughnut specimens, which were compared with the experiment results for FEA validation.

**Figure 4:**
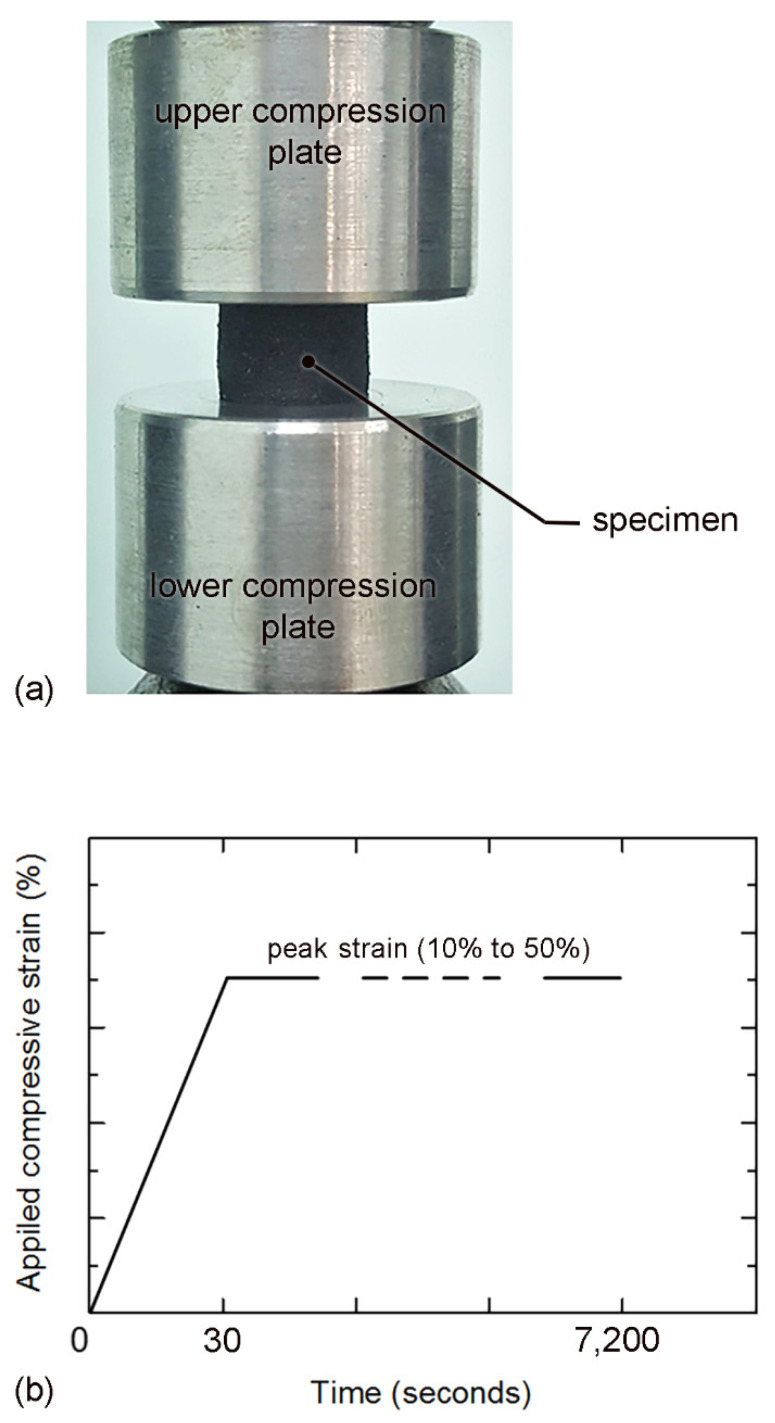
(**a**) Compressive stress relaxation test; and (**b**) applied compressive strain vs. time.

On a universal testing machine (Instron: 5969 with a 10-kN load cell), the stress relaxation test was conducted under 25 °C temperature and 55% relative humidity. Two compression plates (6061 aluminum alloy) with flat surfaces were used as a jig for compression, as shown in Figure 4a. Specimen was compressed to the peak strain within 30 s, and then the compressive strain was maintained for the entire test period; i.e., 120 min (Figure 4b). Counterforce and time were recorded with a 1 Hz sampling rate during the stress relaxation test. Measurement accuracy was 0.01 N in force. Stress relaxation tests were performed at various peak strains (10 to 50% of specimen thickness). In each level of compression, the stress relaxation tests were repeated 3 times, and their average was used for the determinations of the hyperelastic and viscoelastic parameters.

## 5. Finite Element Analysis

Commercial FEA software (i.e., Abaqus/Standard [17]) was applied for the estimation of the material parameters (i.e., the constants of hyperelastic and viscoelastic models) as well as the prediction of stress relaxation of the rubber specimens. The solution of the stress relaxation requires that both the force and moment equilibriums be maintained at all times over any arbitrary volume of the body (i.e., specimen, lower compressive plate, and upper compressive plate). FEA is based on the approximation of the nonlinear equilibrium requirement (i.e., a partial differential equation) by replacing it with a weaker requirement (i.e., the equilibrium is maintained in an average sense over a finite number of divisions of the volume of the body). The exact equilibrium statement is arranged in the form of the virtual work statement (i.e., a weak form of the equilibrium equations), subsequently reduced to the approximate form of equilibrium, and used in a finite element model. Complete theory regarding the FEA can be obtained in [17].

**Figure 5:**
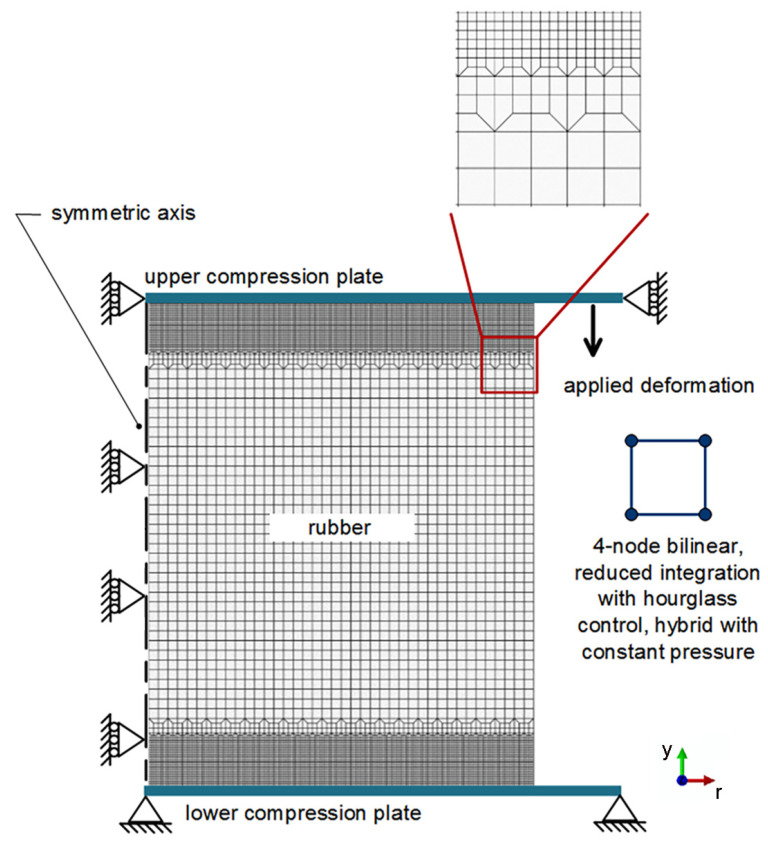
FEA model of the disc specimen.

The material parameters (i.e., the constants of hyperelastic and viscoelastic models) were obtained from the stress relaxation tests of disc-A using the standard specimens recommended by ASTM D6147 [2]. After the estimations of the hyperelastic and viscoelastic parameters, the numerical predictions of rubber behavior with various applied strains and geometries were performed, and then compared against the experimental results (i.e., disc-B and doughnut specimens). For disc specimen, a 2-dimensional FEA (*yr*-plane of cylindrical coordinate) was firstly performed using a half model of the specimen (Figure 5), and then swept around the symmetric axis (*y*-axis of cylindrical coordinate) with 180 steps from 0° to 360° to obtain a complete 3-dimensional FEA model. The fully integrated first-order triangular meshes are usually overly stiff, and exhibit volumetric locking in incompressible problems; e.g., the present rubber. Accordingly, the triangular meshes were not selected for the present FEA. To represent the actual deformation behavior of rubber, 4-node bilinear, reduced integration with hourglass control, and hybrid with constant pressure elements or CAX4RH elements were used for disc and doughnut specimens. Since the modulus of the compression plate (i.e., a 6061 Al alloy with 70-GPa modulus) is significantly higher than that of rubber, the compression plate was assumed to be an infinitely stiff body.

Previously, Suh and Graham [19] studied the apparent modulus (or compression modulus) of a rectangular block of rubber tested up to a 10% strain, whose one surface was bonded to a rigid plate and the other surface in contact with a frictional surface. The 2-dimensional finite element analysis and experimental results for the rubber blocks with shape factors ranging from 1 to 6 were compared for the validation of the analytic results. They found that friction coefficient plays an important role in the design characteristics of the rubber block. Accordingly, in the present work, the frictional contact and master–slave algorithm were applied to the contacts between the compression plates and rubber. Surfaces of the compression plate were the master surfaces, while surfaces of rubber were the slave surfaces. Slip of rubber was assumed to occur when shear stress is greater than the critical shear stress (*τ_c_*); i.e.,

(10) $$\tau \geq \tau_{c}=\mu \sigma_{n}\;$$

where *σ_n_* is the normal stress, and *μ* is the Coulomb friction coefficient. Based on ASTM G115 [20], the Coulomb friction coefficient at the interface between the compression plate and rubber specimen was evaluated using a tribometer (CSM: TRB-S-DE). The friction coefficient (*μ*) is 0.24.

The boundary condition (i.e., Dirichlet boundary condition) was prescribed along the symmetry axis, the lower compressive plate, and the upper compressive plate (Figure 5). On the lower compression plate, the displacement in all directions and the rotation around all axes of lower compression plate were constraint. In turn, only displacement in the *y* direction was allowed for the upper compression plate. On the symmetric axis of the rubber specimen, only displacement in *y* direction was allowed. Previously, Sorvari et al. [21] found that the assumption of an infinite small ramp time, i.e., Heaviside step function or unit step function, can induce severe errors. Accordingly, the multiple steps of applied compressive strain were applied for the present FEA. Compressive strain was gradually increased with intervals of 30 steps from zero to the peak strain, while the stress response of each step of applied strain was calculated. Subsequently, the peak strain was maintained for the entire test period; i.e., 120 min. During this period, the stress responses were calculated with 60 steps of FEAs.

Dependence of FEA result on element size was minimized by varying element size until variation of the maximum principal stress in rubber is lower than 5%. It was observed that the maximum stress increases with the decrease in element size. Similar FEA modeling was applied for the doughnut specimen, except that the elements around the symmetric axis were removed to generate the hollow space. FEA models of disc-A and disc-B before sweeping around the symmetric axis had the smallest element of 65 μm, and had 5000 and 11,480 elements, respectively. On the other hand, the FEA model of the doughnut had the smallest element of 40 μm, and had 3896 elements.

## 6. Result and Discussion

### 6.1. Hyperelastic Behavior

**Figure 6:**
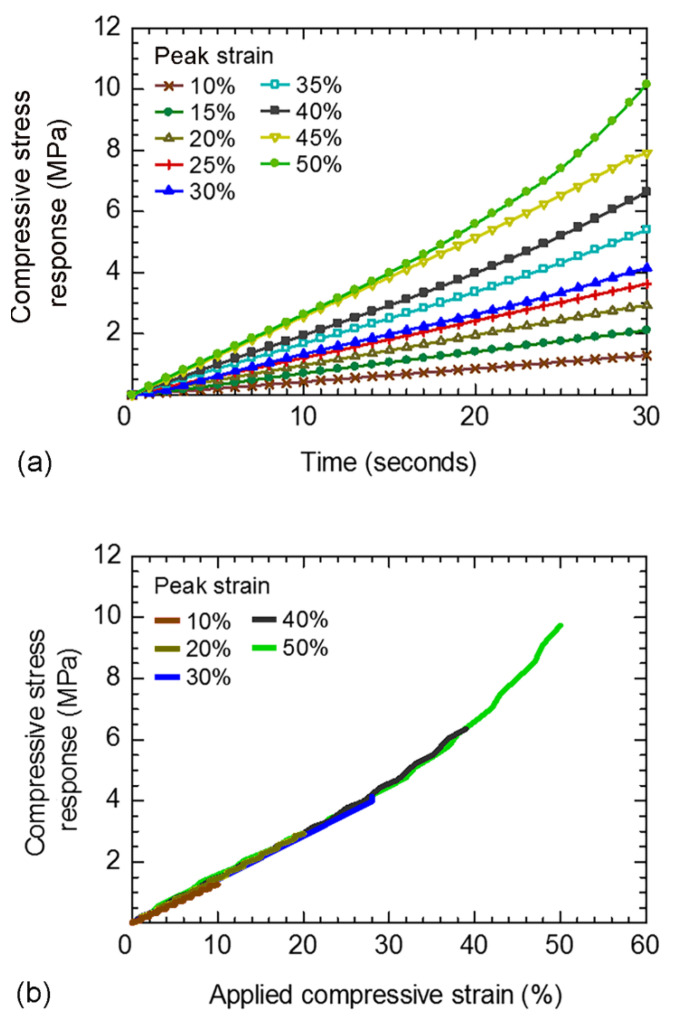
Hyperelastic period of stress relaxation test using disc-A: (**a**) compressive stress response vs. time; and (**b**) applied compressive strain vs. compressive stress response.

Compressive stress responses in a function of time of disc-A during the hyperelastic period of stress relaxation test are shown in Figure 6a. Specimens were compressed to various peak strains (10 to 50%) within 30 s; thus, the strain rates were in the range of 3.3 × 10^−3^ to 1.7 × 10^−2^ s^−1^. Relationships between the applied compressive strain and compressive stress response of disc-A at various peak strains (i.e., various strain rates) are shown in Figure 6b. All plots under various strain rates are nearly on the same line, which indicates the independence of the compressive stress response on strain rate. It is confirmed that the application of compressive strain within 30 s (i.e., ASTM D6147 [2]) can minimize the influence of strain rate, and the hyperelastic behavior can be assumed during periods of applied strain for the present rubber.

**Figure 7:**
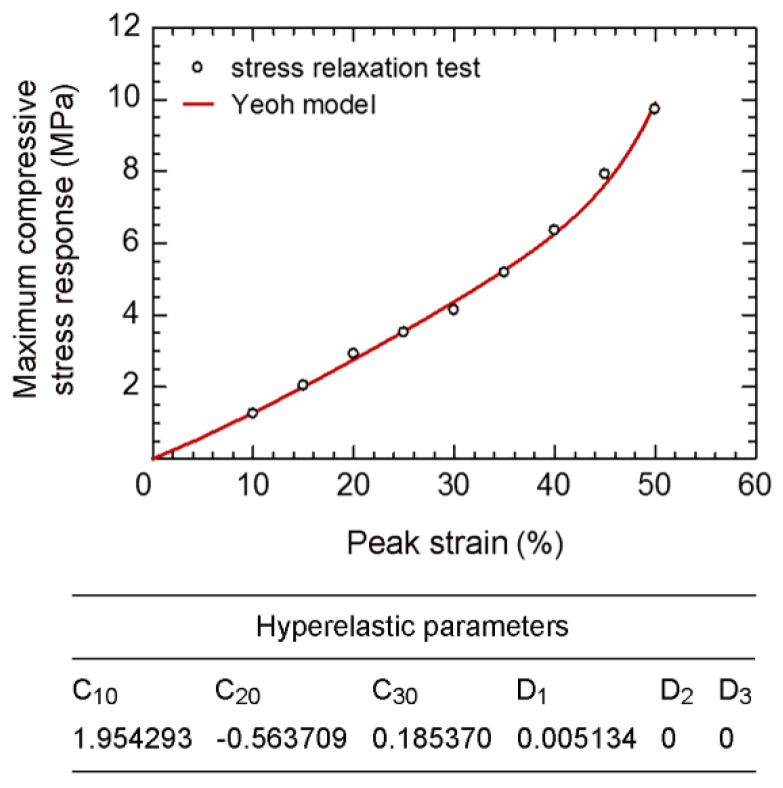
Maximum compressive stress response vs. the peak strain and hyperelastic parameters of disc-A during a hyperelastic period of the stress relaxation test.

The relationship between the peak strain and the maximum compressive stress response at 30 s of disc-A is shown in Figure 7. As a special case of the reduced polynomial model, the Yeoh model (Equation (1)) was applied for the determination of hyperelastic parameters by inserting the stress responses and applied strains in Abaqus. Various numbers of network (*n*) were applied for the parallel rheological framework (PRF). It was found that the PRF with *n* ≥ 3 gives the suitable hyperelastic parameters for the present rubber. Based on the PRF with *n* = 3, the estimated hyperelastic parameters of rubber are shown in Figure 7. To verify the estimated hyperelastic parameters, the relationship between the maximum applied strain and the maximum stress response was calculated using the Yeoh model, and then compared with the experimental results (Figure 7). Differences between the maximum stress responses from the Yeoh model and experiment are marginal; i.e., the highest difference is <5% at the peak strain of 50%. It is confirmed that these hyperelastic parameters were correctly estimated, and the Yeoh model can be applied for the prediction of the hyperelastic behaviors of the present rubber.

Although the hyperelastic behavior can be successfully assumed for the present rubber under rapid application of compressive strain; it is possible that some rubber components may be used under slower strain rates than that recommended by ASTM D6147. Thus, the assumption of hyperelastic behavior may not be suitable for these rubber components, and the assumption of viscoelastic behavior should be used instead.

### 6.2. Viscoelastic Behavior

**Figure 8:**
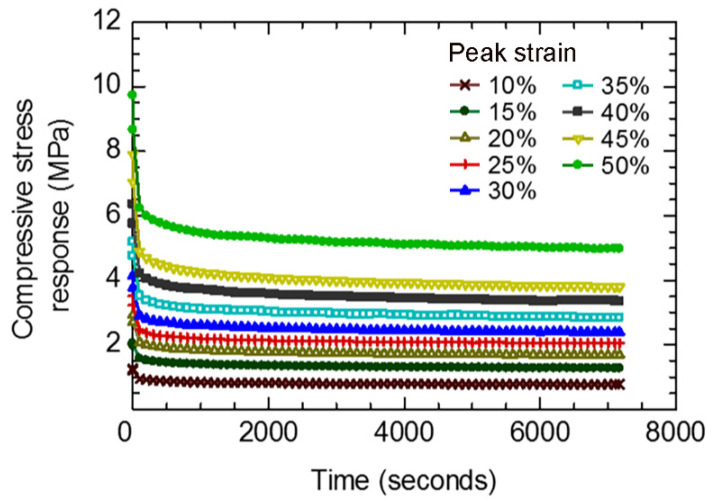
Compressive stress response vs. time of disc-A during viscoelastic period of stress relaxation test.

**Figure 9:**
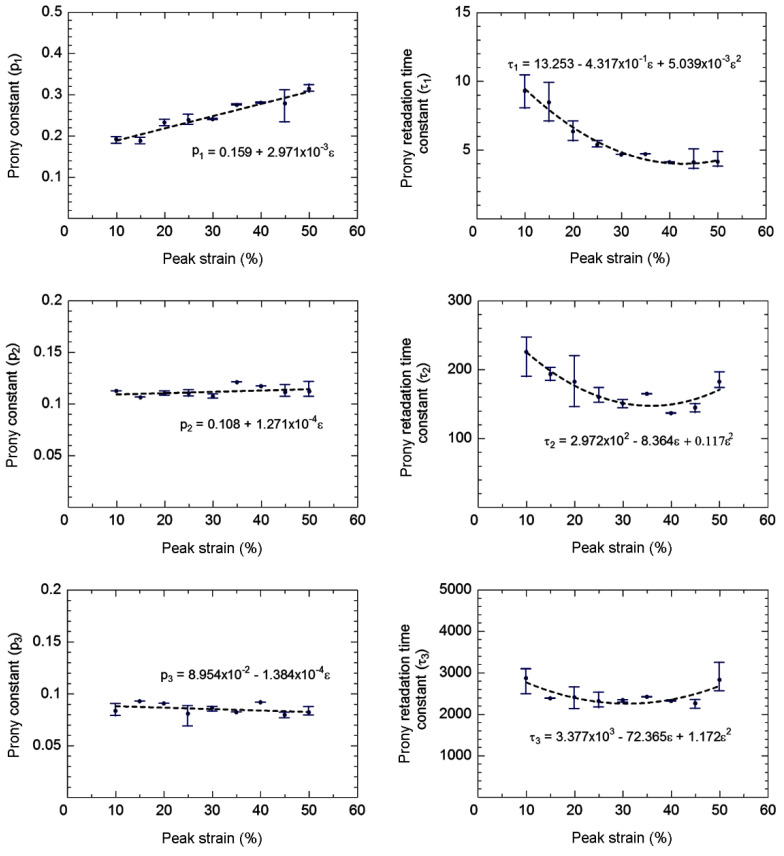
Estimated viscoelastic parameter vs. peak strain of disc-A.

Compressive stress responses of disc-A during the viscoelastic period as a function of time are shown in Figure 8. Relaxation of the compressive stress response with time is observed during the initial stage, becoming stable after approximately 500 s. As a viscous response of three viscoelastic networks (*n* = 3), the Prony series (Equation (7)) was applied for the determination of the viscoelastic parameters by inserting the compressive stress responses as a function of time in Abaqus. The estimated viscoelastic parameters of the rubber for each peak strain are shown in Figure 9. Equations representing the variations in the viscoelastic parameters with peak strain are shown in Figure 9, which can be used for the estimation of the viscoelastic parameters of rubber between 10% to 50% peak strain.

**Figure 10:**
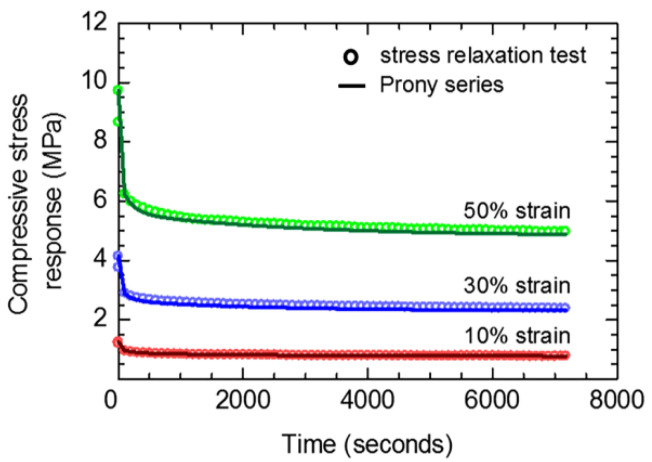
Comparisons between compressive stress responses of disc-A during viscoelastic period estimated from Prony series and those of experiments.

To verify the estimated viscoelastic parameters, the compressive stress responses during the viscoelastic period as a function of time were estimated using the Prony series, and then compared with the experimental results (Figure 10). It is noted that the stress relaxation in a cross-linked rubber can be shown on a double logarithmic representation [11]. However, the present stress relaxations are presented on a plot of linear stress vs. linear time for better comparison. Differences between the compressive stress responses from the Prony series and experiment are marginal; i.e., the highest difference is <5% at the peak strain of 50%. It is confirmed that the viscoelastic parameters using the parallel rheological framework (PRF) with *n* = 3 were correctly estimated the viscoelastic behavior of the present rubber. The higher numbers of network (*n* > 3) have been applied in the PRF to estimate the viscoelastic behavior; however, they provided an insignificant difference when compared to the PRF with *n* = 3.

### 6.3. Numerical Prediction of Stress Relaxation Behavior

**Figure 11:**
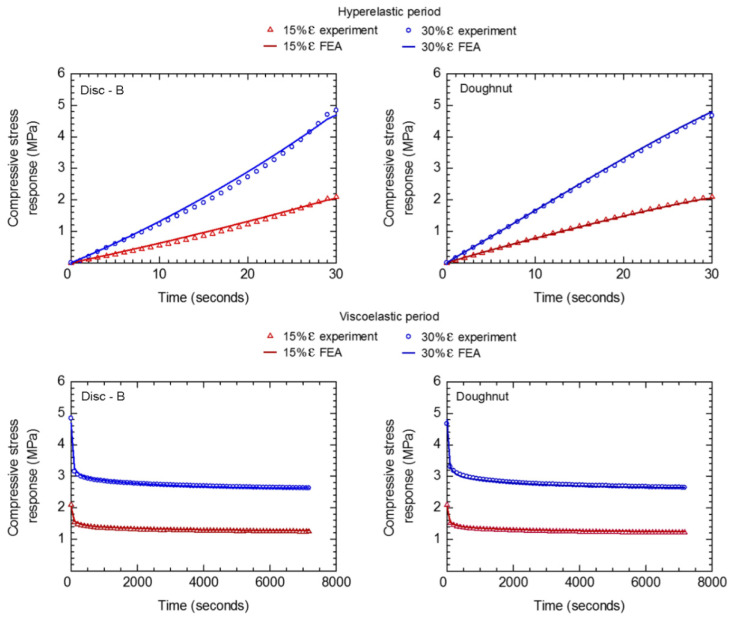
Comparisons between stress relaxation behaviors estimated from the FEAs and those from experiments.

The hyperelastic and viscoelastic parameters estimated from the stress relaxation test of disc-A were used as material input for FEA to predict the compressive stress response of disc-B and doughnut under various applied strains. Subsequently, the FEA results were compared against the experimental results, as shown in Figure 11. For disc-B and doughnut specimens, the predicted compressive stress responses during hyperelastic and viscoelastic periods are in good agreement with the experimental results. The maximum differences in compressive stress response between the FEAs and experiments are lower than 5%. It is confirmed that the stress relaxation behavior of rubber can be numerically estimated based on the assumptions of the Yeoh model and Prony series.

### 6.4. Deformation along the Contact between the Compression Plate and Rubber

The stress distribution in rubber cannot be measured directly from the stress relaxation test. Thus, the numerical calculation of localized stresses during hyperelastic and viscoelastic periods was performed using FEA. However, it is believed that the stress distribution in rubber can be affected by the rubber deformation along contact between the compression plate and rubber [19]. Therefore, the changes in contact radius of disc-A in a function of compressive strain were measured during the stress relaxation experiment, and compared with those of the FEA prediction.

**Figure 12:**
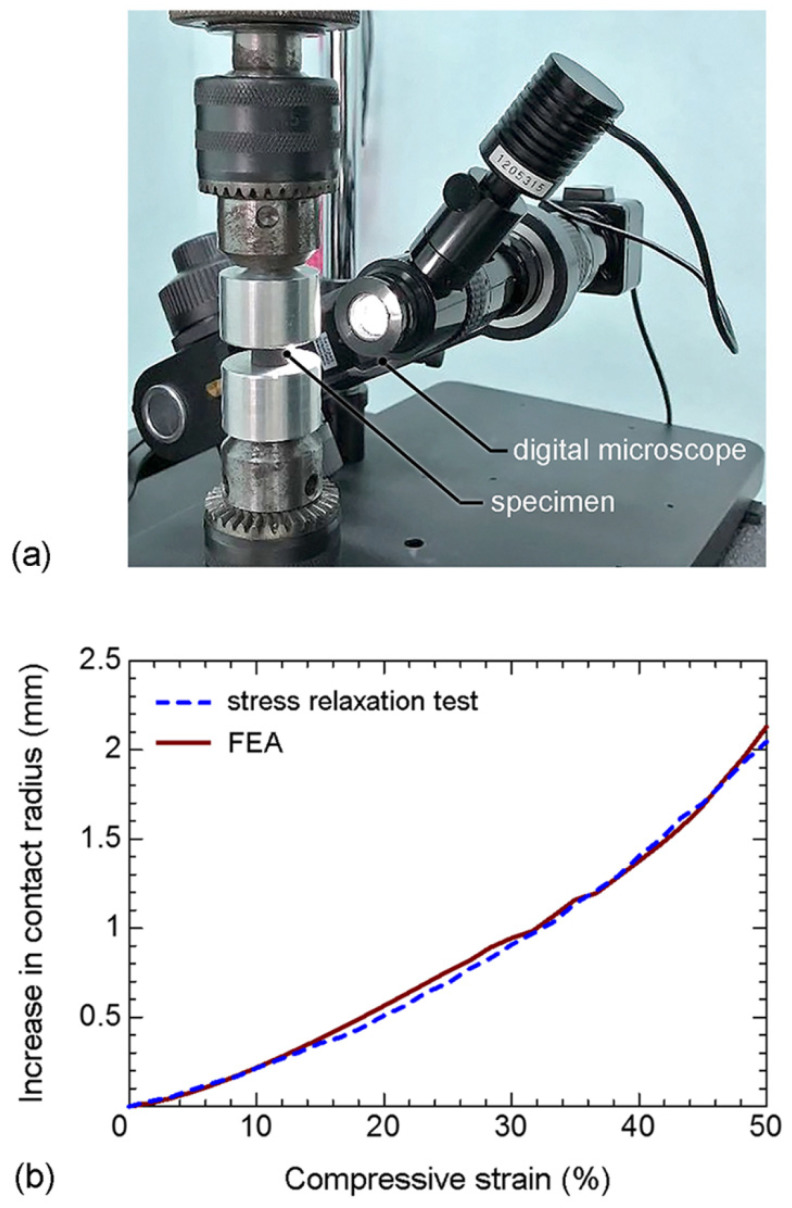
(**a**) Measurement of an increase in the contact radius of disc-A during the stress relaxation experiment; and (**b**) increases in the contact radius of disc-A during stress relaxation experiment (50% peak strain).

During the stress relaxation experiment of disc-A at a 50% peak strain, a digital microscope (Shodensha: Z200PC3 with image analysis software) was used to capture the image of contact edge every 1 s. An increase in the contact radius of disc-A was measured with an accuracy of 10 μm. Measurement of an increase in the contact radius of disc-A during the stress relaxation experiment is shown in Figure 12a. During hyperelastic period, the contact radius of disc-A increases with compressive strain until 50% peak strain, and becomes stable when the compressive strain is maintained constant during the viscoelastic period. Increases in the contact radius of disc-A during the stress relaxation experiment at 50% peak strain are shown in Figure 12b. The experiment and FEA results are in good agreement; i.e., the rubber deformation along the contact between the compression plate and rubber is correctly taken into consideration for FEA. Therefore, FEA can be applied for the calculation of localized stress in rubber.

### 6.5. Localized Stress during the Hyperelastic Period

**Figure 13:**
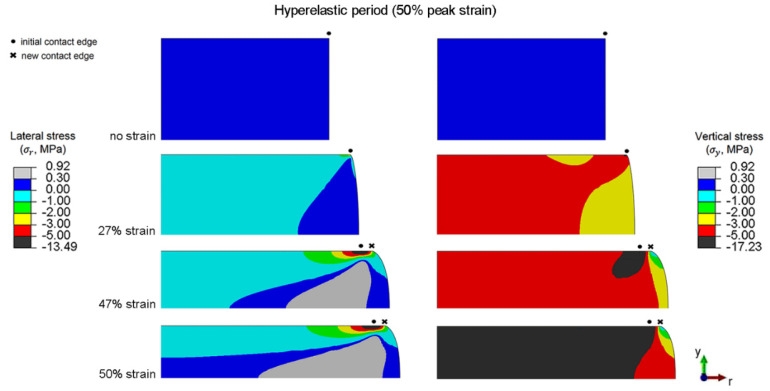
Distributions of the lateral and vertical stresses of disc-A during the hyperelastic period (50% peak strain).

Distributions of the lateral and vertical stresses during the hyperelastic period of disc-A under the peak strain of 50% are shown in Figure 13. The initial contact edge is defined as the contact edge at 0% strain. Lateral and vertical stresses are significantly high near the contact edge, and increase with compressive strain. The influence of rubber deformation along the contact between the compression plate and rubber on the lateral and vertical stress distributions can be observed especially near the contact edge. Slipping of the initial contact, formation of new contact, and slipping of the new contact are possible along the contact during hyperelastic period. Details of these mechanisms are discussed as follows:

(i) *Slipping of initial contact*. At the beginning of the applied strain (0 to 27% strain), the compressive force and friction force at the contact are low; thus, the constraint in lateral expansion of rubber along the contact is marginal. Accordingly, the slipping of the initial contact is possible.
(ii) *Slipping of initial contact, and formation of new contact*. The friction force at the contact increases with the compressive deformation, and eventually the friction force is high enough to constrain the slipping of the initial contact. As the applied strain is continued, another mechanism occurs to support the large compressive deformation; i.e., the rubber near the contact edge starts to flow into contact, and forms the new contact. At intermediate compressive strains (27 to 47% strain), the increase in the contact radius of disc-A is the combination between the slipping of the initial contact edge and the formation of a new contact.
(iii) *Slipping of initial contact, formation of new contact, and slipping of new contact*. Although friction force increases with compressive deformation, the friction force also decreases with the increase in contact area. At a high compressive strain (47 to 50% strain), the decrease in friction force becomes the dominating mechanism; thus, the slipping of the initial contact and slipping of the new contact are possible again.

### 6.6. Localized Stress during the Viscoelastic Period

**Figure 14:**
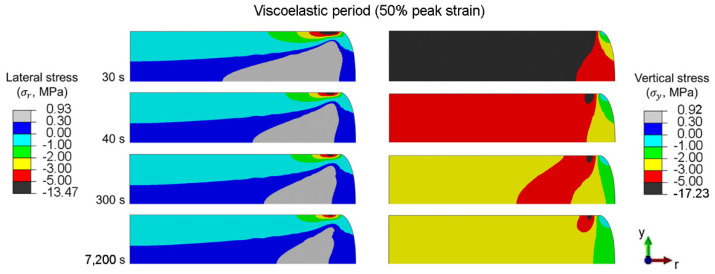
Distributions in the lateral and vertical stresses of disc-A during the viscoelastic period (50% peak strain).

Distributions of the lateral and vertical stresses during the viscoelastic period of disc-A under the peak strain of 50% are shown in Figure 14. Lateral and vertical stresses are significantly high along the contact between the compression plate and disc-A, and decrease with time. Although the stress distribution in rubber can be affected by rubber deformation along the contact between the compression plate and rubber, the contact radius of disc-A remains constant during the viscoelastic period. Under viscoelastic behavior, the strain energy of rubber can be released by the movement of molecular structures; i.e., the viscous effect of rubber. The release in strain energy causes a reduction in the compressive stress at contact, which consequently reduces the friction force at the contact. However, at 50% peak strain, the remaining friction during the viscoelastic period is too high; therefore, the reversed slipping of the contact and the reversed flow of rubber out of the contact are unlikely.

Release in strain energy occurs rapidly during the beginning of the viscoelastic period (30 to 300 s), and becomes relatively slow after approximately 500 s. Eventually, the release in strain energy becomes stable. Because the relaxation in vertical stress involves the release in strain energy from the movement of molecular structures, it mainly occurs in the bulk of the rubber. On the other hand, the relaxation in lateral stress mainly occurs around the contact because it involves the decrease in friction force at contact.

Based on the findings, the hyperelastic and viscoelastic behaviors of rubber involve not only the response of its molecular structure but also the behavior at the contact. The bulk stress relaxation as well as the localized stress relaxation of rubber during hyperelastic and viscoelastic periods can be numerically calculated. These findings provide some guidelines for engineers to (i) calculate the localized stress response of rubber during rapid application of compressive strain and stress relaxation; and (ii) experimentally and numerically evaluate the influence of applied strain, geometry, and frictional contact on the stress relaxation behavior of rubber and viscoelastic polymeric components. Moreover, the present findings can be integrated into research works regarding the mechanical system, such as a Bayesian approach (i.e., a probabilistic method used to estimate the unknown parameters, and subsequently predicting the characteristic output), to estimate the material parameters for propagating fractures in elastic solids [22,23].

## 7. Conclusions

In accordance with ASTM D6147, compressive stress relaxation tests of rubber at various strains (10% to 50%) were performed, and the hyperelastic and viscoelastic parameters were evaluated using the Yeoh model and Prony series, respectively. Finite element analysis (FEA) models were used for the predictions of the compressive stress responses of rubber. After the validation with the stress relaxation test of rubber, it was confirmed that the present FEA successfully gave the predictions of compressive stress relaxation. The results are summarized as follows:

(1) Application of compressive strain within 30 s (i.e., ASTM D6147) could minimize the influence of relaxation, and the hyperelastic behavior could be assumed during a period of applied strain for the present rubber. The hyperelastic behavior of rubber was successfully characterized by the Yeoh model. In turn, the viscoelastic behavior of rubber was successfully characterized by the Prony series.
(2) Hyperelastic and viscoelastic parameters estimated from the stress relaxation test of disc-A were used as material input for FEA to predict the compressive stress response of disc-B and doughnut under various applied strains. The predicted compressive stress responses during the hyperelastic and viscoelastic periods were in good agreement with the experimental results.
(3) During the hyperelastic period of the rubber disc tested at 50% peak strain, the lateral and vertical stresses were significantly high near the contact edge, and increased with compressive strain. An influence in rubber deformation along the contact between the compression plate and rubber on the lateral and vertical stress distributions was observed especially near the contact edge. Mechanisms of rubber deformation along the contact between the compression plate and rubber were the slipping of the initial contact, formation of a new contact, and slipping of the new contact.
(4) During the viscoelastic period of the rubber disc tested at 50% peak strain, the lateral and vertical stresses were significantly high along the contact between the compression plate and rubber, and decreased with time. High friction along the contact prohibited the reversed slipping of the contact and the reversed flow of rubber out of the contact; thus, the contact radius of the rubber disc remained constant during the viscoelastic period.

## Figures and Tables

**Table 1:** Dimensions of the rubber specimen.

Scheme	Geometry (mm)			Initial Shape Factor
	*D*	*D* *_o_*	*D* *_i_*	
disc-A	12.7	-	-	0.40
disc-B	25.0	-	-	0.78
doughnut	-	25.0	12.7	0.38

Note: thickness (*t*) is 8 mm.
